# Evolving strategies for addressing CAR T-cell toxicities

**DOI:** 10.1007/s10555-024-10227-1

**Published:** 2024-12-15

**Authors:** Alexander W. Rankin, Brynn B. Duncan, Cecily Allen, Sara K. Silbert, Nirali N. Shah

**Affiliations:** 1 Pediatric Oncology Branch, Center for Cancer Research, National Cancer Institute, National Institutes of Health, Bethesda, MD 20892 USA; 2 Division of Hematology, Department of Medicine, Johns Hopkins University, Baltimore, MD USA; 3 Department of Critical Care Medicine, National Institutes of Health, Bethesda, MD USA

**Keywords:** CAR T-cell, B-cell acute lymphoblastic leukemia, B-cell lymphoma, Resistance, Relapse

## Abstract

The field of chimeric antigen receptor (CAR) T-cell therapy has grown from a fully experimental concept to now boasting a multitude of treatments including six FDA-approved products targeting various hematologic malignancies. Yet, along with their efficacy, these therapies come with side effects requiring timely and thoughtful interventions. In this review, we discuss the most common toxicities associated with CAR T-cells to date, highlighting risk factors, prognostication, implications for critical care management, patient experience optimization, and ongoing work in the field of toxicity mitigation. Understanding the current state of the field and standards of practice is critical in order to improve and manage potential toxicities of both current and novel CAR T-cell therapies as they are applied in the clinic.

## Introduction

**Fig. 1:**
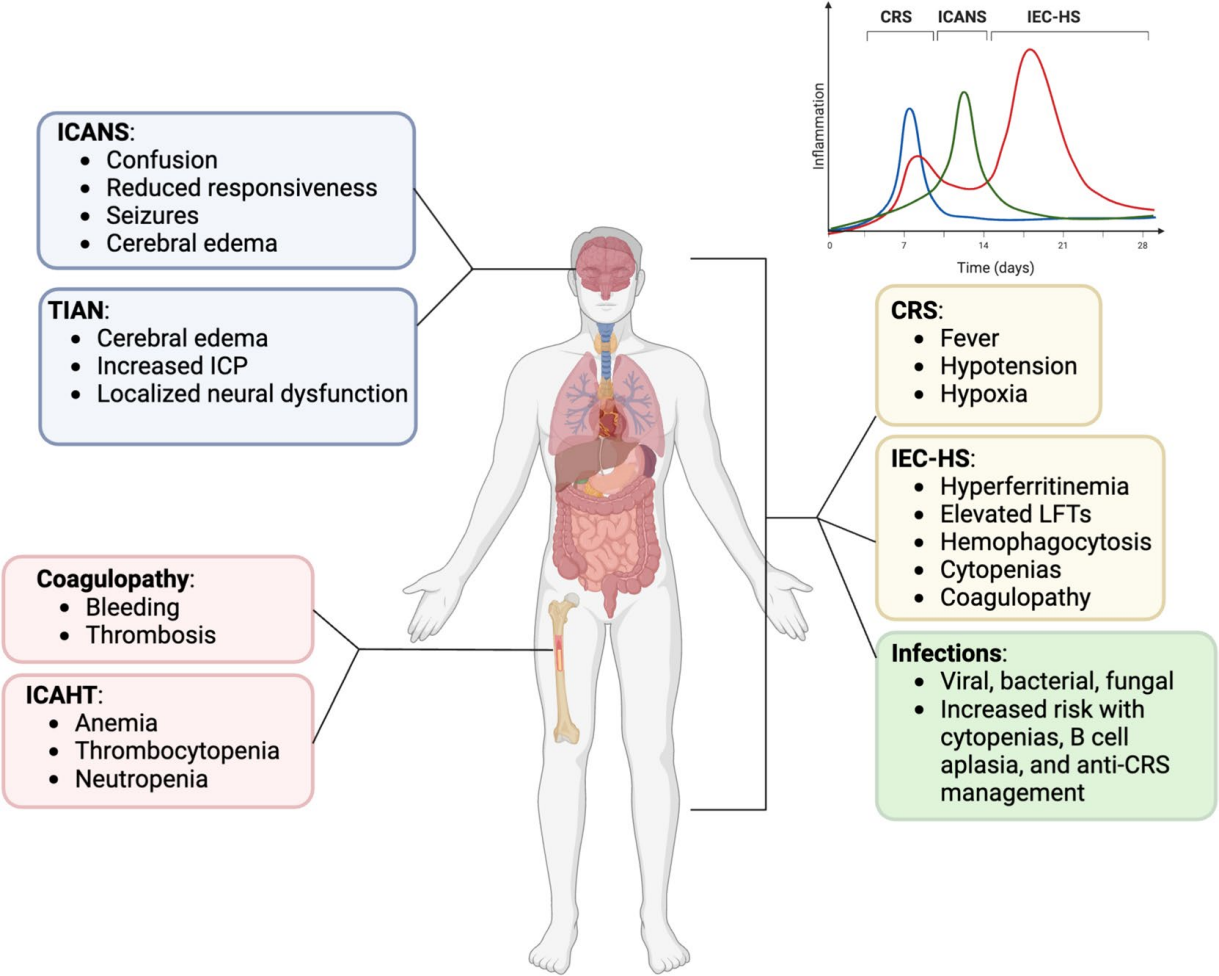
Whole body effects of CAR T-cell toxicities. CAR T-cell therapy-related toxicities have systemic effects with overlapping clinical manifestations and timelines. Toxicities in the same colors affect similar organ systems and may be difficult to distinguish from one another. CRS, IEC-HS, and infections often exert more systemic clinical effects. Time of onset (inset graph) may help distinguish between similar toxicities. Created with BioRender.com

Chimeric antigen receptor (CAR) T-cells have been a revolutionary gene therapy for the treatment of hematologic malignancies, including leukemia, lymphoma, and myeloma. While CAR T-cells have had a good deal of success against these diseases, they have also demonstrated a wide array of toxicities, ranging from mild to life-threatening. Furthermore, as more CARs have come through the clinic, the field has witnessed a wider range of side effects. Some of these appear to be specific to certain CAR constructs whereas others might be disease- or patient-specific. After their introduction to the clinic in the early 2000s, the field of CAR therapies has blossomed in the hopes of applying this treatment modality to other conditions, including solid tumors and autoimmune diseases. Undoubtedly, these new applications and targets will unveil unforeseen toxicities as a result of CAR T-cell treatment. In this review, we aim to call attention to some of the most clinically salient side effects of this therapy (Fig. 1) while drawing attention to specific considerations for the critical care provider as well as the overall patient experience. The field continues to evolve, not only in CAR engineering but also in terms of toxicity characterization, mitigation, and research. We conclude by highlighting the current state of the field in this regard, which includes both clinical and preclinical advances in toxicity mitigation.

## Overview of toxicities associated with *CAR* T-cell therapy

### Cytokine release syndrome (CRS)

CRS has become a well-described complication of CAR T-cells since the first infusion in 2003 [1]. CRS is a supraphysiologic immune response resulting in elevations in serum levels of interleukin (IL)-6, interferon γ (IFNγ), and other proinflammatory cytokines along with clinical signs of fever, hypotension, capillary leak, and/or end organ dysfunction [2]. Symptoms range from mild to life-threatening, and a universally accepted grading system from the American Society for Transplantation and Cellular Therapy (ASTCT) has helped to standardize diagnosis and management (Table 1). These standards have allowed for a streamlined comparison of rates and severity of CRS between different constructs across clinical trials and in the real-world setting. For example, Bachy et al. compared axicabtagene ciloleucel (axi-cel) to tisagenlecleucel (tisa-cel) for diffuse large B-cell lymphoma (DLBCL), noting low-grade CRS was seen more frequently with axi-cel compared to tisa-cel but higher-grade CRS did not differ significantly between the two [3]. Emergence of real-world data is important in elucidating how the toxicity landscape may change with changes in populations treated and management strategies. Rates of CRS in patients receiving axi-cel or brexucabtagene autoleucel (brexu-cel) have largely reflected those seen in the pivotal clinical trials (Table 2) [4–7]. Additionally, early real-world experience with tisa-cel for B-cell acute lymphoblastic leukemia (B-ALL) and non-Hodgkin lymphoma (NHL) demonstrated similar efficacy with improved safety (CRS grade > 3 16% in B-ALL, 4.5% in NHL) compared to the pivotal trials (Table 2) [8].

**Table 1:** Guidelines for the diagnosis and management of CAR-associated toxicities

Toxicity	Grading	Management
Cytokine release syndrome (CRS) Key references [2, 9]	**ASTCT 2019 guidelines** **Grade 1**: ≥ 38 °C, normotensive, no hypoxia **Grade 2**: ≥ 38 °C, hypotension not requiring vasopressors, and/or hypoxia requiring low flow or blow-by oxygen **Grade 3**: ≥ 38 °C, hypotension requiring 1 vasopressor ± vasopressin, and/or hypoxia requiring high flow (> 6 L/min), facemask, nonrebreather, or venturi mask **Grade 4**: ≥ 38 °C, hypotension requiring multiple vasopressors (excluding vasopressin), and/or hypoxia requiring positive pressure (e.g., CPAP, BiPAP, mechanical ventilation)	**First line**: Tocilizumab for adults with ASTCT grade 2 CRS Tocilizumab for children with ASTCT grade 3 or prolonged grade 2 CRS Consider preemptive intervention (i.e., prolonged grade 1) in high-risk cases [10, 11] **Second line**: Any patient unresponsive to 1 dose of tocilizumab should receive steroids with a 2nd dose of tocilizumab **Third line**: Any patient unresponsive after 2 doses tocilizumab and steroids may receive third line agents including anakinra, siltuximab, and high-dose methylprednisolone
Immune effector cell-associated neurotoxicity syndrome (ICANS) Key references [2, 9, 12–14]	**ASTCT 2019 guidelines, patients ≥ 12 years** **Grade 1**: ICE 7–9; awakens spontaneously **Grade 2**: ICE 3–6; awakens to voice **Grade 3**: ICE 0–2; awakens only to tactile stimulus; clinical seizure that resolves or nonconvulsive seizure that resolves with intervention; focal edema **Grade 4**: ICE 0; unarousable, stupor, coma; prolonged seizure (> 5 min) or repetitive seizures without return to baseline; deep focal motor weakness (e.g., hemi/paraparesis); decerebrate/decorticate posturing; CNVI palsy, papilledema, Cushing’s triad, diffuse cerebral edema **ASTCT 2019 guidelines, children < 12 years** **Grade 1**: CAPD score 1–8 **Grade 2**: CAPD score 1–8 **Grade 3**: CAPD score ≥ 9 **Grade 4**: unable to perform CAPD	**Pre-CAR evaluations**: baseline neuro exam, EEG, brain MRI Neurology involvement for patients with neuro comorbidities Preventative medications (e.g., levetiracetam) **Initial interventions**: neuro monitoring (exams, ICE score) days 0–30 or longer if delayed/prolonged toxicity, steroids, reduce neuroexcitotoxic medications (e.g., cefepime, imipenem) **Refractory**: continue/increase steroids, consider addition of anakinra, thiamine repletion, siltuximab If no cerebral edema, consider CSF removal ± IT steroids **Signs of cerebral edema**: maintain normotension, normothermia, normocarbia, aggressive seizure control, elevate head of bed, hypertonic saline, pentobarbital coma
Movement and neurocognitive treatment-emergent adverse events (MNTs)		Enhanced bridging therapy to reduce MM burden Handwriting assessments for early symptom detection Early and aggressive CRS/ICANS treatment [15]
Infections Key references [16]	Grading per CTCAE for study reporting	**Prophylaxis** **Bacterial**: fluoroquinolones for ANC < 0.5 × 109/L **Viral**: acyclovir or valacyclovir, from LD to 6 months post-CAR **Fungal**: fluconazole or micafungin for ANC < 0.5 × 10^9^/L through ANC recovery **PJP**: TMP-SMX or alternative (atovaquone, pentamidine, dapsone) **General management** Follow institutional fever and neutropenia guidelines* Early and frequent infectious disease consultation
B-cell aplasia		**Immunoglobulin replacement**: consider if IgG < 400 mg/dL
Immune effector cell-associated HLH-like syndrome (IEC-HS) Key references [9, 17–19]	**2020 SITC guidelines** **Grade 1**: asymptomatic or mild symptoms not requiring intervention **Grade 2**: mild to moderate symptoms requiring intervention (e.g., immunosuppressive agents for IEC-HS, transfusions for hypofibrinogenemia) **Grade 3**: severe or medically significant but non-life threatening (e.g., coagulopathy requiring transfusions, hospitalization for new AKI, respiratory distress) **Grade 4**: life threatening; intervention indicated (e.g., severe bleeding or hypotension, respiratory failure, dialysis)	**First line**: high-dose steroids and anakinra **Second line**: ruxolitinib **Third line**: emapalumab or etoposide **T-cell depleting therapies**: etoposide as a one-time dose; it is an established therapy for primary and secondary HLH, preferred over other more immunosuppressive agents (e.g., alemtuzumab) **Adjuncts**: consider recombinant thrombopoietin and G-CSF for associated cytopenias
Immune effector cell-associated hematotoxicity (ICAHT) Key references [20, 21]	**EHA/EBMT consensus guidelines** **Day 0 to day + 30 post-CAR (early)**: **Grade 1**: ANC ≤ 500/µL for < 7 days **Grade 2**: ANC ≤ 500/µL for 7–13 days **Grade 3**: ANC ≤ 500/µL for ≥ 14 days OR ANC ≤ 100/µL for ≥ 7 days **Grade 4**: ANC never above 500/µL OR ANC ≤ 100/µL for ≥ 14 days **Day > 30 post-CAR (Late)**: **Grade 1**: ANC ≤ 1500/µL **Grade 2**: ANC ≤ 1000/µL **Grade 3**: ANC ≤ 500/µL **Grade 4**: ANC ≤ 100/µL	**Pre-CAR evaluations**: replace myelosuppressive drugs **Early (day 0 to 30)**: supportive blood product transfusions Antimicrobial prophylaxis G-CSF if ANC < 500/mm^3^ by day + 10, avoid in ongoing CRS **Prolonged (> 30 to < 90 days)**: rule out alternative causes (active primary disease, secondary myeloid malignancy) G-CSF for prolonged severe neutropenia, infections BM studies if not responsive to G-CSF Eltrombopag or romiplostim for patients at risk of bleeding **Late (> 90 days)**: continue antimicrobial prophylaxis, evaluate alternative causes Consider allogeneic HCT in complete aplastic anemia Consider immunosuppression in LGL
Coagulopathies Key references [17, 22]	Grading per CTCAE for study reporting	**Bleeding**: transfusions, factor replacement, vitamin K **Thrombosis**: anticoagulation (UFH, LMWH, oral agents), site-directed thrombolysis
Tumor infiltration-associated neurotoxicity (TIAN) Key references [23, 24]	**Mahdi et al. proposed grading scale**^†^ **Grade 1**: headaches with fevers OR worsening existing neuro signs/symptoms causing minor functional deficits **Grade 2**: moderate changes in neuro exam that substantially affect function **Grade 3**: severe neuro clinical signs/symptoms that may affect cardiorespiratory functions OR Signs/symptoms of increased ICP (> 20 mmHg) responsive to intervention **Grade 4**: life threatening, elevated ICP (> 20 mmHg) refractory to CSF drainage, warranting urgent neurosurgical intervention (e.g., EVD, VPS) OR Signs/symptoms of impending herniation OR Severe medullary dysfunction requiring airway protection and/or mechanical ventilation	**Pre-CAR evaluations**: consider Ommaya reservoir placement in patients with tumors in high-risk locations (e.g., posterior fossa) **Type 1**: *Global mechanical factors* CSF diversion, corticosteroids, hyperosmolar therapy for obstructive hydrocephalus or peritumoral edema **Type 2**: *Localized inflammation* Conservative management with observation and supportive care often sufficient for higher brain structures Intensive care (intubation, ventilation) and pharmacologic intervention may be required if lower brainstem or cervical spinal cord function involvement

*CAR*, chimeric antigen receptor T-cell; *ASTCT*, American Society for Transplantation and Cellular Therapy; *CPAP*, continuous positive airway pressure; *BiPAP*, bilevel positive airway pressure; *ICE*, immune effector cell-associated encephalopathy score; *CAPD*, Cornell Assessment for Pediatric Delerium; *EEG*, electroencephalogram; *MRI*, magnetic resonance imaging; *CSF*, cerebrospinal fluid; *IT*, intrathecal; *MM*, multiple myeloma; *axi-cel*, axicabtagene ciloleucel; *tisa-cel*, tisagenlecleucel; *BCMA*, B-cell maturation antigen; *cilta-cel*, ciltacabtagene autoleucel; *ide-cel*, idecabtagene vicleucel; *CTCAE*, Common Terminology Criteria for Adverse Events; *ANC*, absolute neutrophil count; *LD*, lymphodepletion; *TMP-SMX*, trimethoprim-sulfamethoxazole; *IgG*, immunoglobulin G; *HLH*, hemophagocytic lymphohistiocytosis; *SITC*, Society for Immunotherapy of Cancer; *AKI*, acute kidney injury; *G-CSF*, granulocyte-colony stimulating factor; *ULN*, upper limit of normal; *EHA*, European Hematology Association; *EBMT*, European Society for Blood and Marrow Transplantation; *HCT*, hematopoietic stem cell transplant; *LGL*, large granular lymphocytic leukemia; *B-ALL*, B-cell acute lymphoblastic leukemia; *B-NHL*, B-cell non-Hodgkin lymphoma; *ICP*, intracranial pressure; *EVD*, external ventricular drain; *VPS*, ventriculoperitoneal shunt

^*^Cefepime is independently associated with neurotoxicity in the context of older age and acute kidney injury[25]

^†^Grading does not distinguish between types 1 and 2 TIAN

**Table 2:** FDA-approved CAR T-cell products

Name	Costim. domain	FDA-approved indications	Low-grade CRS (1–2)	High-grade CRS (3–4)	Low-grade neurological events (1–2)^‖^	High-grade neurological events (3–4)^‖^	Pivotal trials
Tisagenlecleucel Other names: Kymriah	4-1BB	- Patients up to 25 years with R/R B-ALL (2nd or greater relapse) - Adults with R/R large B-cell lymphoma (LBCL) - Adults with R/R follicular lymphoma after ≥ 2 lines of therapy	30–57%	22–25% 0% in FL 5.2% BCL	21–40%^‡^	12–13%	ELIANA (B-ALL) [26] ELARA (FL) [27] BELINDA (BCL, not FDA approved) [28] JULIET (LBCL) [29]
Axicabtagene ciloleucel Other names: Yescarta KTE-C19	CD28	- Adults with R/R LBCL o Refractory to 1st line or relapse within 12 months of 1st line o R/R to 2nd line - Adults with R/R FL after ≥ 2 lines of therapy	75–84%	6–13%	37–40%	19–28%	ZUMA-1 (LBCL) [30] ZUMA-5 (FL, MZL) [31]^#^ ZUMA-7 (LBCL) [32]
Brexucabtagene autoleucel Other names: Tecartus KTE-X19	CD28	- Adults with R/R mantle cell lymphoma (MCL)	76% MCL	15% MCL	32% MCL	31% MCL	ZUMA-2 (MCL) [33] ZUMA-3 (B-ALL) [34]
		- Adults with R/R B-ALL	65% ALL	24% ALL	35% ALL	26% ALL	
Lisocabtagene maraleucel Other names: Breyanzi JCAR017	4-1BB	- Adults with R/R LBCL o Refractory to 1st line or relapse within 12 months of 1st line ± HSCT eligible o R/R to 2nd line	40–48%	1–2%	7–26%	4–10%	TRANSCEND (LBCL) [35] TRANSFORM (LBCL) [36] PILOT (LBCL) [37]
Idecabtagene vicleucel Other names: Abecma bb2121	4-1BB	Adults with R/R multiple myeloma after 4 or more prior lines of therapy	83–84%*	5%	12–15%	3%	KarMMa [38] KarMMa-3 [39]
Ciltacabtagene autoleucel Other names: Carvykti JNJ-68284528	4-1BB	Adults with R/R multiple myeloma after 4 or more prior lines of therapy	63–90%	1–4%	4.5–14%^◆^	0–2%^◆^	CARTITUDE-1 [40] CARTITUDE-4 [41]

Abbreviations: *FDA*, US Food and Drug Administration; *CRS*, cytokine release syndrome; *R/R*, relapsed/refractory; *B-ALL*, B-cell acute lymphoblastic leukemia; *FL*, follicular lymphoma; *BCL*, B-cell lymphoma; *MZL*, marginal zone lymphoma; *HSCT*, hematopoietic stem cell transplant

^*^Any CRS

^‡^Neurologic event any grade

^◆^Specified ICANS

^#^Toxicities reported in aggregate for all treated patients, not separated by disease type

‖Most studies report neurologic events rather than ICANS since ICANS was not formally defined when many trials were conducted

CRS occurs as CAR T-cells expand in response to antigenic stimulus and ignite an inflammatory cascade. The backbone of management aims to suppress those signals using tocilizumab, an IL-6 receptor inhibitor that is FDA-approved for the treatment of CRS, and corticosteroids [42]. These agents can reverse symptoms when implemented early [10] or potentially prevent onset when used prophylactically [43], and despite one report to the contrary [44] have generally not been found to negatively influence CAR T-cell function overall [45–48]. Despite efficacy in low-grade CRS, repeated dosing of tocilizumab for refractory CRS fails to add much benefit and is generally discouraged after two doses [49]. High-risk or treatment-refractory patients often require escalating doses of corticosteroids and potentially alternative therapies as discussed below (see Sect. 5) [49]. As more CAR products with unknown toxicity profiles enter the clinic, creative strategies to optimize safety may be required, such as fractionating CAR T-cell dosing [50].

### Immune effector cell-associated neurotoxicity syndrome (ICANS)

Neurological toxicities following CAR T-cell therapy are common. ICANS is a constellation of acute symptoms following CAR T-cell infusion ranging from headache and mental status changes to seizures, coma, and cerebral edema [2]. ICANS onset often follows CRS (Fig. 1) but is CAR- and disease-dependent [12], with resolution typically occurring within 4 weeks for CD19-targeted CAR T-cells [51, 52]. Moreover, many patients embark upon CAR T-cell therapy with neurologic comorbidities (i.e., prior central nervous system (CNS) radiation, methotrexate toxicity), which may predispose to complications. Incidence of ICANS in the real-world has not significantly deviated from rates seen in large clinical trials for axi-cel and brexu-cel (Table 2) [53]. In DLBCL, axi-cel is associated with substantially higher rates of ICANS compared to tisa-cel [3].

Thorough pre-treatment evaluations, including baseline neurological exams, neuroimaging, prophylactic antiepileptics, and other medication optimization, are recommended prior to initiating therapy when possible [12] (Table 1). Tocilizumab for isolated ICANS is discouraged since tocilizumab does not cross the blood–brain barrier and causes a transient elevation in serum and cerebrospinal fluid (CSF) IL-6, which may exacerbate neurotoxicity [54]. Corticosteroids, on the other hand, have been effective first-line agents for high-grade ICANS and are often initiated early in high-risk patients [9, 49]. Many other agents are being evaluated for both prophylaxis and treatment of ICANS (see Sect. 5). While neuroimaging is important for optimal management, findings often lag behind clinical signs [12], and standard neurological critical care guidelines should be followed for any patient demonstrating signs of cerebral edema (see Sect. 3). Notably, some of the most severe cases of ICANS have been associated with rapid CAR expansion, sharp increases in cytokines, and breakdown of the blood–brain barrier [12, 55, 56]. Fortunately, with early and aggressive medical management, most cases of ICANS are reversible.

As more patients receive CAR T-cells and novel products enter the clinic, new toxicities can be expected to emerge. For example, transverse myelitis has been reported in a handful of patients [55, 57], and some have experienced irreversible paralysis or ongoing deficits [13, 58, 59]. Another notable entity is delayed neurotoxicity. While most ICANS occurs in the first few weeks following infusion, symptoms can recur months later, potentially due to CAR re-expansion and cytokine production [60, 61]. Extended neurological monitoring for ≥ 3 months is recommended following B-cell maturation antigen (BCMA)-targeted CAR therapy, despite these CARs having lower rates of ICANS overall, to screen for movement and neurocognitive treatment-emergent adverse events (MNTs) [12, 62] (Table 1). This specific toxicity is characterized by Parkinsonism-like symptoms, including bradykinesia, flat affect, rigidity, gait disorder, and micrographia [12], and is hypothetically due to on-target/off-tumor targeting within the basal ganglia. MNTs are reported in 1–5% of ciltacabtagene autoleucel (cilta-cel) patients [12, 15], but unlike ICANS, standard therapies are often insufficient to reverse symptoms.

### Infections

Many patients receiving CAR T-cells are heavily pre-treated with reduced immune reserve, which is further compromised by lymphodepletion, anti-inflammatory medications for CRS/ICANS, and post-treatment cytopenias [16]. It is unclear whether different CAR constructs themselves increase infection risk, but rates of CRS, ICANS, and duration of B-cell aplasia likely play a role.

Screening and antimicrobial prophylaxis remain the backbone of infectious risk reduction and are recommended prior to embarking on CAR T-cell therapy (Table 1) [16]. B-cell aplasia post-CD19 CAR T-cells may last months, reducing humoral immunity. Plasma cells, which are long-lived antibody-producing cells that often express lower CD19 than other B-cells, occasionally survive CD19 CAR T-cell therapy but are often very sensitive to BCMA CAR T-cells [63, 64]. In children with developing immune systems and fewer plasma cells at baseline, CD19 CAR T-cells may deplete enough plasma cells to induce increased susceptibility to infection compared to adults receiving CD19-targeting therapy. IgG replacement for patients with severe hypogammaglobulinemia (i.e., IgG < 400 mg/dL) is often recommended either via intravenous or subcutaneous replacement strategies [63], but risks, benefits, and costs should be weighed for each patient.

Post-CAR T-cell infections are common (up to 55% of patients within the first 1–2 years, 33% with grade ≥ 3) [26, 29, 30, 65]; however, fatal infections remain rare. Most infections occur within the first 2 weeks and are typically bacterial, but respiratory viral infections occur frequently as well. Viral reactivation of herpes simplex virus (HSV) or varicella-zoster virus (VZV) can occur due to lapses in viral prophylaxis, and fungal infections are documented in up to 8% of patients [66]. Distinguishing between infection and CRS can be difficult, so both entities should be evaluated for and treated concurrently. As a general rule, early and frequent involvement of infectious disease experts is prudent in CAR T-cell patients, and our institutional practice is to seek an infectious disease consultation for any patient proceeding to CAR T-cells.

### Immune effector cell-associated HLH-like syndrome (IEC-HS)

IEC-HS is a unique hyperinflammatory syndrome that is distinct from CRS and is characterized by many of the clinical and laboratory findings of hemophagocytic lymphohistiocytosis (HLH). It often appears as CRS is resolving or has completely resolved [17]. Hyperferritinemia is a diagnostic requirement, but relying on specific levels as cutoffs should not delay clinical intervention when IEC-HS is otherwise suspected even if ferritin is below a defined threshold. Treatment is based off of classic HLH mitigation strategies [67] as well as therapies for CRS and other IEC-associated syndromes [68] (Table 1). Currently, there are no established preemptive treatment strategies for IEC-HS. Corticosteroids and anakinra are first line for IEC-HS with consideration of second- and third-line agents (i.e., ruxolitinib, emapalumab) in more severe or refractory cases [17, 18]. The goal of therapy is to minimize inflammation while avoiding abrogation of CAR T-cells if possible. Single-dose etoposide, an established therapy in primary and secondary HLH, may be used if IEC-HS becomes life-threatening [17]. Furthermore, the importance of supportive measures including aggressive blood product support, coagulopathy management, and infection prevention and treatment cannot be overstated.

### Immune effector cell-associated hematotoxicity (ICAHT)

The most commonly reported adverse event following CAR T-cell therapy is hematologic toxicity. Lymphodepletion commonly contributes to early cytopenias, and although recovery is often rapid, it is not always observed [20]. Neutrophil recovery is usually biphasic, but some patients develop an “aplastic” phenotype that stretches over months [69]. The underlying etiologies of ICAHT are multifactorial with potential contributors including infection, medications and chemotherapy, immune-mediated stem cell or mature blood cell destruction, and infiltrative bone marrow processes (i.e., relapse or secondary malignancies) [69].

Early ICAHT management includes transfusion support, antimicrobial prophylaxis, and granulocyte-colony stimulating factor (G-CSF) for severe neutropenia or infection (Table 1). While G-CSF has not demonstrated an association with increased high-grade CRS/ICANS [70], granulocyte–macrophage colony-stimulating factor (GM-CSF) should be avoided as this cytokine is implicated in CRS and ICANS and may exacerbate those processes [71]. Patients with refractory or prolonged ICAHT may benefit from thrombopoietin agonists [72] or autologous or allogeneic hematopoietic stem cell boost if available [73]. In the case of severe, life-threatening ICAHT, allogeneic hematopoietic stem cell transplant (HCT) is a last resort to restore normal hematopoiesis that must be carefully considered and weighed against the possibility of spontaneous count recovery [74].

### Coagulopathy

Coagulopathy is another common side effect of CAR T-cells, presenting as bleeding and/or thrombosis [22] (Table 1). It has been observed at variable rates in association with different CAR products, in the setting of IEC-HS [75], and with disease involvement of different anatomical sites. A report of CD19 CAR T-cells for adults with lymphomatous GI involvement described significant bleeding in 42.3% [76]. Variable bleeding rates have been observed with different CAR constructs, with 30% of patients experiencing clinically significant bleeding after an investigational CD22-targeting CAR that was associated with lower-grade CRS but a greater degree of hypofibrinogenemia (< 150 mg/dL) [77] vs. 45.6% after tisa-cel which was associated with higher-grade CRS on the ELIANA and ENSIGN trials [78]. Patients are at increased risk for disseminated intravascular coagulation (DIC), exhibiting laboratory derangements, and clinical manifestations such as thrombosis, bleeding, and/or multiorgan failure. Disturbances in coagulation parameters often correlate with CRS severity [22, 79]. Addressing coagulopathy in patients receiving CAR T-cells centers on blood product and factor optimization, with concurrent CRS-directed therapies when indicated [54]. Maintaining fibrinogen of > 150 mg/dL during CRS is recommended, with frequent monitoring since fibrinogen may be artificially elevated in the setting of CRS prior to coagulopathic consumption [54, 78]. Platelet transfusion thresholds may differ by institution, but generally a goal of > 20 × 10^9^/L is implemented during CRS [78, 80]. It is also important to consider that some patients may be on anticoagulation for an active clot and develop subsequent CAR-related coagulopathy. In those treated with direct oral anticoagulants (DOACs), it would be prudent to transition to readily reversible agents such as enoxaparin or even a heparin drip prior to treatment with CAR T-cells in the event that they become coagulopathic or profoundly thrombocytopenic. To date, there are no universal guidelines for the management of these patients, and it remains an area for further study.

### Tumor infiltration-associated neurotoxicity (TIAN)

The CNS is anatomically unique, sheltered by both the blood–brain barrier and the bony skull and vertebrae. Primary brain tumors, CNS lymphoma, and CNS metastasis from solid tumors pose unique challenges for cellular therapies. TIAN, which has only recently been defined, is observed following CAR T-cell therapy for CNS tumors and can be primarily mechanical, inflammatory edema and neurological symptoms secondary to increased intracranial pressure, or more localized neural dysfunction specific to the area of immunotherapy-related inflammation [23] (Table 1). How TIAN manifests is highly dependent on tumor location, with tumors of the thalamus and brainstem conferring much higher risk of poor outcomes [24]. Further, the method of CAR T-cell delivery may influence both efficacy and toxicity. In a trial of intraventricular CAR T-cells targeting EGFR and IL13Rα2, all six patients developed early and moderate to severe neurotoxicity that included symptoms of both ICANS and TIAN [81]. Given early signs of clinical efficacy of some CNS tumor-directed constructs, TIAN may become a more common CAR-mediated toxicity as cellular therapy advances in the treatment of CNS tumors. It can be difficult to differentiate TIAN from ICANS; however, management has significant overlap. In a clinical study of GD2-targeted CAR T-cells for diffuse midline gliomas, TIAN was anticipated to arise frequently, and a standard management algorithm consisted of CSF removal, hypertonic saline, anti-cytokine agents, and steroids [24]. Another strategy, intrathecal steroid administration, may provide benefit to patients with ICANS or TIAN who are refractory to systemic steroids without impacting CAR T-cell efficacy [14]. Better understanding of the pathophysiology of TIAN and how best to manage it is needed as more CAR T-cell therapies directed at CNS tumors come to clinic.

### On-target/off-tumor toxicity considerations

As additional CAR constructs make their way to the clinic, history will continue to be an important teacher. The success of CD19-targeted CAR T-cells stems in large part from the relatively tolerable on-target/off-tumor toxicity of B cell aplasia, which is not a fatal complication and can be treated with immunoglobulin replacement therapy. Vital healthy tissues often express antigens that are targeted by CAR therapies, as is the case for CD33 and CD123, which are expressed on both acute myeloid leukemia and healthy myeloid progenitors and could lead to significant hematotoxicity and bone marrow aplasia if targeted by CAR T-cells [82, 83]. Many strategies are currently under investigation in both preclinical and early clinical trials in the hopes of minimizing these toxicities and are discussed in more detail later in this review. On-target/off-tumor toxicities are perhaps most troublesome when treating solid tumors. Not only have there been severe manifestations of on-target/ off-tumor toxicities during preclinical development [84], but cases have also occurred in clinical trials as well [85, 86]. In one trial of an anti-mesothelin T-cell receptor fusion construct for mesothelin-expressing tumors, grade 3 or higher pneumonitis was seen in 16% of patients as well as 3 grade 5 events related to the T-cell therapy (bronchoalveolar hemorrhage, pneumonitis, and respiratory failure in the setting of CRS) [87]. In another well-known example, two patients died after receiving TCR T-cells targeting MAGE-A3 for myeloma and melanoma due to cardiogenic shock that developed secondary to T-cells recognizing an unrelated peptide on cardiomyocytes [88]. Thus, this highlights the importance of thorough preclinical modeling while acknowledging that there is no substitute for human trials when it comes to determining CAR T-cell safety.

### Toxicity risk factors

Experience treating patients with hematologic malignancies with CAR T-cells over the last decade has elucidated numerous factors with clear impact on outcomes and toxicities (Table 3) [89, 90]. While post-infusion cytokines and biomarkers are associated with toxicities [91–93], the focus here will be on pre-treatment risk factors with potentially actionable interventions and/or prognostic value.

**Table 3:** Risk factors for the development of CAR-associated toxicities

Baseline risk factor	Evidence-based impacts on toxicity
**Disease burden**	
Bone marrow disease/lymphomatous disease	Higher disease burden is correlated with increased incidence and severity of CRS, ICANS, and IEC-HS [86–92]
Non-CNS extramedullary disease	Site-specific toxicities, i.e., eyelid swelling associated with an orbital mass [87, 93–95]
CNS disease	Conflicting reports on impact on ICANS with studies reporting no association between CNS disease and ICANS [96] and others reporting increased incidence and severity of ICANS in those with CNS disease [97, 98]
**Baseline lab values**	
Platelets	Thrombocytopenia has been associated with severe CRS and ICANS [99], IEC-HS [92], as well as increased risk of bleeding events [22]
CRP	Elevated CRP has been associated with severe CRS and ICANS [99] and IEC-HS [92]
Platelets, CRP and LDH	The modified EASIX score = (LDH × CRP)/Platelets is associated with severe CRS and ICANS [99]
Ferritin	Elevated ferritin has been associated with IEC-HS [92]
vWF and Ang2:Ang1	Markers of endothelial activation found to be associated with CRS severity [84]
Platelets, hemoglobin, ANC, ferritin, CRP	Together thrombocytopenia, anemia, neutropenia, elevated ferritin, and CRP contribute to the CAR-HEMATOTOX score; a score ≥ 2 puts patients at risk for severe prolonged neutropenia and infections [21, 100]
**CAR construct and/or preparation regimen**	
T-cell dose	Higher dose associated with increased risk of CRS [84]
Bulk CD8 + T-cells without central memory selection	Associated with increased risk of CRS [84]
CD4/8 selection	Associated with increased risk of IEC-HS [101]
Costimulatory domain	CD28-containing CARs typically inducing earlier CRS onset with increased low-grade CRS compared to 4-1BB constructs; incidence of higher-grade CRS appears stable across costimulatory domains [102]
Fludarabine	Increased risk of CRS when incorporated into lymphodepletion regimen [84]
**Demographics**	
Age	Mixed results in studies of older adults; one study reports no difference in rates of severe CRS or ICANS [103], another reports higher rates of severe ICANS in patients ≥ 65 years [104] Infants have comparable rates of CRS, ICANS, and infections as older children, but higher rates of prolonged cytopenias [105, 106]
Ethnicity	Hispanic patients have been found to have higher rates of severe CRS [107]

Abbreviations: *CAR*, chimeric antigen receptor; *CRS*, cytokine release syndrome; *ICANS*, immune effector cell-associated neurotoxicity syndrome; *ICE-HS*, immune effector cell-associated hemophagocytic lymphohistiocytosis-like syndrome; *CNS*, central nervous system; *CRP*, C-reactive protein; *LDH*, lactate dehydrogenase; *EASIX*, Endothelial Activation and Stress Index; *vWF*, von Willebrand factor; *Ang*, angiopoietin; *ANC*, absolute neutrophil count

Disease burden at the time of CAR T-cell infusion is arguably the most critical toxicity biomarker, with higher marrow, lymphomatous, or plasma cell disease demonstrating clear negative associations with response and toxicities [21, 94–99]. Non-CNS extramedullary disease (EMD) in B-ALL has also been associated with poor response and may lead to site-specific toxicities [95, 100–102]. Although it would follow that active CNS leukemia would contribute to ICANS risk, data are conflicting, suggesting ongoing need to research this association [103–105].

Specific baseline lab values, including thrombocytopenia and elevated C-reactive protein (CRP), have been associated with CRS and ICANS on a univariate level and in combination with LDH in the modified EASIX (Endothelial Activation and Stress Index) score for adult patients with B-ALL and DLBCL [106]. Similarly, elevated baseline CRP and ferritin, along with decreased platelet and neutrophil counts, have been correlated with risk of IEC-HS [21]. Endothelial activation markers, including elevated von Willebrand factor (vWF) and angiopoietin 2:angiopoietin 1 ratio pre-infusion, have been associated with increased CRS severity in adults with B-cell malignancies [92]. Baseline cytopenias and inflammation (elevated CRP and ferritin) together as part of the CAR-HEMATOTOX score have been associated with prolonged severe neutropenia and infections following CAR T-cell infusion in DLBCL and multiple myeloma (MM) patients [107, 108]. Further, bleeding events have been observed more often in patients with baseline thrombocytopenia and in association with high-grade ICANS [22].

Characteristics intrinsic to the CAR product and preparative regimen also contribute to toxicities. In one CD19 CAR T-cell study, higher T-cell dose and bulk CD8^+^ T-cell manufacturing without central memory cell selection were both associated with increased CRS [92]. The same study found that fludarabine as part of lymphodepletion also increased CRS risk and that higher CAR T-cell expansion correlated with CRS severity [92]. A trial of CD22 CAR T-cells in pediatrics and young adults with B-ALL demonstrated that CD4/8 selection contributed to increased incidence of IEC-HS, prompting dose de-escalation [109]. CAR costimulatory domains also influence toxicity profiles, with CD28-containing CARs typically inducing earlier CRS onset with increased low-grade CRS compared to 4-1BB constructs, whereas incidence of higher-grade CRS appears stable across endodomains [110].

Associations between patient demographics and post-CAR toxicities have been observed, but the data are mixed. One study demonstrated that patients > 75 years receiving commercial CAR T-cells for NHL and MM had neither worse rates nor higher grades of CRS or ICANS compared to those 65–74 years [111]. Another study of axi-cel and tisa-cel demonstrated that while older patients (65–83 years) had comparable rates of high-grade CRS, they experienced twice the risk of high-grade neurotoxicity when compared to a younger cohort (19–64 years) [112]. In pediatrics, two studies comparing outcomes of infants treated with tisa-cel demonstrated comparable or lower rates of CRS, ICANS, and infections and a higher incidence of prolonged cytopenias compared to the ELIANA registration trial, which excluded infants [113, 114]. Faruqi et al. found that pediatric B-ALL patients with Hispanic heritage were more likely than white non-Hispanic patients to experience grade > 3 CRS after adjusting for disease burden, with similar patterns described in patients with NHL and MM [115]. In the same study, race had no association with other toxicities; however, the majority of patients were non-white Hispanic and Hispanic, limiting analysis. Lastly, obesity, which is associated with chemotherapy resistance and poorer overall survival in B-ALL [116, 117] and increased mortality in NHL [118], has not been associated with an increased risk of CRS and/or neurotoxicity to date [115, 119].

### Prognostication and predictive models

Predicting a patient’s risk for severe toxicities is important. Multiple IEC options with different risk profiles exist, so calculating accurate risk projections allows providers to plan for early risk mitigation and prophylactic strategies. With a more complete understanding of these risks, the patient-provider team can prioritize therapies with toxicity profiles most aligned to the patient’s goals of care. In allogeneic HCT, the hematopoietic cell therapy comorbidity index (HCT-CI) was established and validated to help predict post-HCT outcomes including non-relapse mortality and overall survival [120, 121]. This model relies on assessment of 17 upfront comorbidities. Despite similarities with HCT, this index has failed to demonstrate predictive utility in a CAR T-cell cohort with B-ALL [122]. The CAR T-cell therapy-specific comorbidity index (CT-CI) is adapted from the HCT-CI, and preliminary data demonstrates the ability to predict overall survival but not toxicities in adults with LBCL [123]. Another tool, “Severe4,” uses a modified version of the Cumulative Illness Rating Scale (CIRS) and has predictive benefit for both progression-free and overall survival as well as severe (grade > 3) CRS in adults with DLBCL [124]. Neither model has been tested in pediatric subgroups or other hematologic malignancies.

Others have looked beyond comorbidities to develop prognostic models from baseline biomarkers. Two particular models have demonstrated validity in predicting post-CAR toxicities. The modified Endothelial Activation and Stress Index (m-EASIX) score was developed from the EASIX score (LDH*Creatinine/Platelets), which correlates endothelial damage with allogeneic HCT outcomes. The m-EASIX (LDH*CRP/Platelets) was applied to adults with B-ALL and DLBCL with higher scores significantly associated with development of severe CRS and ICANS at specific timepoints, as well as reduced odds of overall response [106]. Lastly, as alluded to previously, the CAR-HEMATOTOX score is a validated model in DLBCL and MM using patients’ baseline hematopoietic reserve and underlying inflammation to assign a score predicting the risk of severe prolonged neutropenia (ANC < 500/mcl for > 14 days), prolonged hospitalization, and infection [107, 125].

## The role of critical care in *CAR*-T cell therapy

Many severe sequelae of CAR T-cell therapy such as hypotension, heart failure, hypoxemia, or neurotoxicity necessitate escalation of care and admission to the intensive care unit (ICU). While often a direct consequence of the CAR T-cells themselves, these issues can also arise from a number of alternative underlying causes (i.e., infection). In patients who have received CAR T-cells, hypotension and hypoxemia in particular require simultaneous treatment with both CAR-specific and more generalized therapies (i.e., antibiotics, diuretics, vasopressors). There must be a low threshold for early evaluation by intensivists, especially for patients at high risk for developing severe toxicities and for patients refractory to initial toxicity interventions.

### Pre-treatment evaluations

Given the unique and potentially severe toxicities of CAR T-cells, it is crucial to understand patients’ baseline functional status and assess for comorbid medical conditions prior to starting therapy. Eligibility criteria for clinical trials are variable but generally strict, often limiting enrollment to younger patients with limited comorbidities (i.e., good performance status, limited end organ disease) [126]. In clinical practice however, CAR T-cells are routinely administered to complex patients, which can cause uncertainty in predicting outcomes. Comorbidities as assessed by CIRS have not been predictive of the risk of ICU admission in DLBCL patients who received commercial CAR T-cells [11]. However, another study found that Eastern Cooperative Oncology Group (ECOG) performance status was independently associated with mortality in adult ICU patients with DLBCL, MM, or B-ALL who had received commercial CAR T-cell products [127].

Patients who receive CAR T-cells are at risk for cardiac and pulmonary complications that requires thoughtful planning before infusion [128]. Cardiac-specific pre-administration evaluations should include an electrocardiogram, echocardiogram, and in certain populations, a baseline troponin [128]. From a pulmonary perspective, pre-administration evaluations should include thoracentesis if pleural effusion is present. For those with underlying pulmonary disease, there is no clear guidance on pre-administration pulmonary function testing and it is therefore not routinely performed [128]. A thorough understanding of baseline cardiac and pulmonary function aids in discussions with patients about potential complications should they have baseline organ dysfunction. In the context of CRS, patients may develop pulmonary edema or pleural effusions, which are localized inflammatory states that may require more urgent management in the ICU. Additionally, in those with pulmonary-based disease, local inflammation may also be problematic in addition to that resulting from CRS alone [101, 129]. Specific management of these complications is not standardized but is largely focused on reducing the inflammatory cytokines associated with the underlying inflammatory process in addition to aggressive supportive management. However, when CRS or other post-CAR T-cell toxicities arise in high-risk cardiac or pulmonary patients, the differential should remain broad. Moreover, in the case of hypotension, both vasodilatory distributive shock and cardiogenic shock should be considered, especially in patients with a prior history of cardiac comorbidities or other pertinent risk factors [128].

### ICU-specific management considerations

While toxicities such as CRS are often reversible, aggressive support may be required to achieve optimal outcomes [130]. Thus, CAR T-cell therapy and intensive care have been closely intertwined from the beginning. Early trials in pediatric B-ALL cite ICU admissions of 15–47% with patients requiring vasopressors (25%), mechanical ventilation (13%), and renal replacement therapy (9%) [94, 131, 132]. Similar rates have been observed in adults [133]. Although many patients treated with CAR T-cells require intensive care, outcomes for the patients admitted to the ICU are similar to those observed in non-ICU patients [134]. Yet despite advances in toxicity mitigation strategies, the number of patients requiring ICU admission will continue to be significant [135]. Combinatorial strategies implementing targeted therapies for CAR-mediated toxicities (i.e., tocilizumab and steroids for CRS) with higher-level support (i.e., vasopressors for hypotension) has allowed for improved care and outcomes for patients receiving CAR T-cells.

Infection is common in the setting of CAR-T cell therapy, with confirmed infections observed in up to 33% of patients admitted to the ICU [134]. Initial presentation can be similar to the vasodilatory state of CRS, thus empiric antimicrobial coverage is critical while collecting data to support a given diagnosis. Infections in this setting are most commonly bacterial but can include invasive fungal and systemic viral infections [134]. Patients may present with isolated hypotension, hypoxemia, or tachycardia without a fever given concomitant steroids and/or tocilizumab [2]. If patients with CRS fail to respond to CRS management alone, special attention to infectious etiologies should be considered.

During the hyperinflammatory states of CRS and/or IEC-HS, fluid management is an individualized process. The ASTCT defines CRS grade based on the degree of hypotension and the requirement for intravenous fluids or vasopressors, highlighting variability in the volume of fluids that can safely be administered. When hypotension is present in the setting of CRS or sepsis, early ICU involvement is required to determine optimal hemodynamic support. Though there are no randomized controlled trials comparing fluids versus early vasopressors, certain clinical indicators (i.e., new cardiomyopathy with rising oxygen requirements) lend themselves to early vasopressor initiation. Norepinephrine is the preferred first-line agent for hypotension requiring pressors in the setting of CRS [2]. In cancer patients in general, mechanical ventilation is associated with higher mortality as compared to noninvasive ventilation, but those patients who deteriorate on noninvasive ventilation and later require intubation have a mortality rate as high as 80% [136]. This is likely rooted in prior data demonstrating improvement in respiratory status with early initiation of continuous positive airway pressure (CPAP) and inferior outcomes in patients who had hypoxemic respiratory failure and underwent mechanical ventilation, with no difference in outcomes between early vs. late intubation [137, 138].

Severe ICANS is another CAR-related toxicity that often requires ICU admission. Cerebral edema is a rare but life-threatening complication of ICANS, which is managed by attempting to reduce intracranial pressure by elevating the head of the bed and administering osmotic agents (i.e., hypertonic saline), steroids, and antiepileptics [12, 139]. Magnetic resonance imaging (MRI) findings in ICANS can be variable and can range from normal to characteristic T2/FLAIR hyperintensities to rare reports of ischemic or hemorrhagic strokes in severe cases [139]. For patients requiring intubation in the setting of cerebral edema, it is imperative to choose sedatives that minimize intracranial pressure, such as propofol, rather than those that may raise it, such as high-dose opiates [99, 140].

### Pediatric-specific considerations

Grading schemas and medical management for ICU patients experiencing CAR T-cell-related toxicities is mostly comparable between pediatric and adult patients, with some differences [141]. The Pediatric Acute Lung Injury Consensus conference (PALICC) group created specific pediatric definitions for acute respiratory distress syndrome (ARDS) that have been applied to grading of hypoxemia associated with CRS [141]. From a neurological perspective, a scoring system known as the Cornell Assessment for Pediatric Delirium (CAPD) was developed and validated to screen for delirium in pediatric ICU patients and has been used successfully to detect early evidence of ICANS in patients under the age of 12 [2, 142]. Importantly, pediatric caregivers play a crucial role in completion of bedside assessments [141, 143, 144]. Management of toxicities is similar in both children and adults; however, there is need for improved pediatric-specific evidence with ongoing studies to minimize the risk of CRS and ICANS in this population (Table 3) [145].

### Goals of care discussions

Patients receiving CAR T-cell therapy have usually received multiple prior lines of therapy, often resulting in significant cumulative toxicities, and are limited with regard to treatment options. Based on patient experiences, there is a need to increase palliative conversations, identify caregivers, and discuss goals of care preceding CAR T-cell therapy [146]. Therefore, conversations on goals of care and life support that consider the individual patient’s baseline functional status, comorbidities, and risk factors should be approached prior to initiation of CAR T-cells. In general, time-limited trials of higher-level critical care may be prudent for CAR T-cell patients since CAR-related toxicities are often reversible [132].

### Future directions in ICU management

Despite the integral role of the intensive care team in the management of CAR T-cell complications, robust data on optimal management practices and standardized guidelines are lacking, with management approaches varying by institution. Future research should focus on optimizing oxygen support, developing guidelines for optimal hemodynamic management, and pre-emptive goal setting with patients and families ahead of potential complications. Standardization will help establish evidence-based approaches and inform best practices for this unique ICU population [132].

## Optimizing the patient experience

### Patient-reported outcomes

In addition to toxicity and trial-based adverse event monitoring, understanding patients’ subjective experiences is critical to fully address CAR T-cell toxicities. Patient-reported outcomes (PROs) are assessment tools allowing for systematic evaluation of patients’ (and caregivers’) subjective experiences, including physical and psychological symptoms, mood, functioning, and quality of life (QOL). PROs have been successfully measured across multiple CAR T-cell trials for patients with hematologic malignancies [147–153] as well as their caregivers [154]. Studies have generally shown that symptoms and QOL initially decline from baseline post-CAR with improvement over time [148–150]. However, the current approach to PROs varies, with substantial differences in measures employed and timepoints assessed. As such, standardization of prospective PROs would allow for greater cross-trial comparisons. For example, Sidana et al. prospectively evaluated QOL, symptom burden, and cognition across three cohorts undergoing CAR T-cell therapy, autologous HCT, and allogeneic HCT. While all cohorts had similar baseline QOL, CAR T-cell patients experienced less decline compared to HCT patients [150]. Efforts to look beyond the feasibility and outcomes of PRO integration, and determining how to use PRO data in real time to enact changes in supportive care are ongoing [155–157].

### Psychosocial standards of care

While CAR T-cell therapy was initially reserved for relapsed or refractory disease and patients faced an unclear prognosis with a high likelihood of toxicities, with established efficacy CAR T-cell therapies are moving earlier into treatment algorithms. Irrespective of when CAR T-cell therapy is administered, to best support these patients, implementing universal supportive care standards is critical. Steineck et al. emphasized three primary domains of psychosocial care for children receiving CAR T-cell therapy, including psychosocial evaluation and support, clear communication, and symptom management [158]. A systematic approach to address these domains is applicable across disease types and across stages of life, as adults face comparable challenges requiring similar support during CAR T-cell therapy [159]. Additional domains to address include social determinants of health, fertility, long-term side effects, and discussions surrounding advance care planning.

### Financial toxicity

Despite potential efficacy, CAR T-cells remain inaccessible to many patients who might otherwise benefit from them due to their high economic costs. In 2023, commercially available CAR T-cells cost upwards of $400,000, and this price continues to climb [160]. The cost of provision of CAR T-cell therapy is also substantial [161], making access even more challenging for those without insurance [162]. Despite soaring production and administration costs that remain largely opaque due to claims of commercial confidentiality from pharmaceutical companies, developing lower cost yet effective CARs is possible [163]. In fact, by decentralizing CAR T-cell production from industry-specified sites to academic centers in India, the cost of CAR T-cells was able to be reduced to approximately one tenth the price [164]. Reducing financial burden on patients could allow many more people, especially those in lower- and middle-income settings, to access a possible cure.

## Translational investigation into toxicity management

### Preclinical toxicity modeling

Developing novel approaches to mitigating CAR T-cell-associated toxicities requires the use of functional *in vivo* models that allow for the study of CAR T-cell interactions with the host immune system and nonmalignant tissue. Given that no animal model can adequately recapitulate the expression of potential human target antigens on nonmalignant tissues or model the complexity of the intact human immune system, developing representative *in vivo* models of toxicity has been a challenge. However, various models with distinct advantages and disadvantages have served to greatly advance our understanding of CAR T-cell toxicity (Table 4).

**Table 4:** Non-standard agents used for CAR-associated toxicity management

Agent	Mechanism	Evidence
Anakinra	IL-1 receptor antagonist, blocking the action of IL-1α and IL-1β	Murine model showed CRS mediated by IL-6 and IL-1 and can be ameliorated through IL-1 blockade [159] Murine model demonstrated the ability of anakinra to abolish CRS in ICANS after tocilizumab failure [163] Anakinra was safe and associated with lower TRM in refractory CRS/ICAN in a retrospective analysis [165] In a small series, 4/6 patients who received anakinra for high-grade refractory ICANS derived clinical benefit [166] Phase 2 trial of anakinra for ICANS prophylaxis after CD19 CAR for lymphoma; all grade ICANS 19%, no grade 4 or 5 events [167]
Siltuximab	Binds directly to IL-6, prevents IL-6 binding to soluble and membrane-bound IL-6 receptors	First-line siltuximab for CRS was safe and feasible in a retrospective analysis, without increased use of further anti-IL-6 therapy or steroids [168] Retrospective analysis of siltuximab for CRS or ICANS, 36/42 (86%) and 19/28 (68%) had improvement of CRS or ICANS after siltuximab, respectively [169]
Emapalumab	Binds to IFNγ and inhibits interaction with cell-surface receptors	Use of emapalumab in a humanized mouse model showed efficacy in mitigating severe CAR-associated toxicity [170] Pharmacologic blockade or genetic knockout of IFNγ reduced macrophage activation without impairing CAR function in a murine model [171] Case report of severe CRS with HLH-like features refractory to conventional management that showed improvement after emapalumab [172]
Ruxolitinib	Inhibitor of JAK1 and JAK2, thus impeding T-cell proliferation and development mediated through JAK/STAT signaling	Usage extrapolated from data in secondary HLH: • 69% response rate to frontline rituximab monotherapy [173] • 74% [174] and 78% [175] response rate in refractory HLH
Etoposide	Impairs DNA synthesis through inhibition of topoisomerase II	Usage extrapolated from efficacy in primary HLH: • HLH-94: 54% 5-year probability of survival [67] • HLH-2004: 62% 5-year probability of survival [176]
Lenzilumab	Directly binds to GM-CSF and prevents signaling through its receptor	GM-CSF neutralization with lenzilumab reduced CRS and neuroinflammation in a murine model [71] Antibody-mediated neutralization or TALEN-mediated inactivation in CAR T-cells abolished monocyte-dependent release of CRS mediators [177] Ongoing phase 2/3 randomized trial of lenzilumab to decrease toxicity in adults with lymphoma [178]
Dasatinib	Tyrosine kinase inhibitor that mainly targets BCR-ABL, but also several other tyrosine kinases, inhibiting T-cell proliferation	Demonstrated dasatinib was able to halt CAR T-cell cytolytic activity, cytokine production, and proliferation *in vitro* and *in vivo* [179] Observed that dasatinib may act as a reversible safety switch to manage CAR-associated toxicity [180] Case report of refractory grade 3 CRS and grade 4 ICANS successfully managed with dasatinib [181]

*CAR*, chimeric antigen receptor; *IL*, interleukin; *CRS*, cytokine release syndrome; *ICANS*, immune effector cell-associated neurotoxicity syndrome; *TRM*, treatment-related mortality; *IFNγ*, interferon gamma; *HLH*, hemophagocytic lymphohistiocytosis; *JAK*, Janus kinase; *STAT*, signal transducer and activator of transcription; *DNA*, deoxyribonucleic acid; *GM-CSF*, granulocyte–macrophage colony-stimulating factor; *TALEN*, transcription activator-like nuclease

Xenograft mouse models are widely utilized and have been very important in establishing CAR T-cell efficacy, but also have several important limitations. For example, many studies have employed NOD/scid/IL2rγ − / − (NSG) mice, which lack functional T, B, NK, and dendritic cell compartments, as an immunodeficient model [182]. While ideal in many ways for human cell engraftment, immunodeficient models generally do not sufficiently allow for the modeling of the interaction between CAR T-cells and the host immune system [183]. Specifically, the incomplete reactivity between human and mouse cytokines as well as the lack of many human effector cells restricts manifestations of toxicity such as CRS [184, 185]. Despite these challenges, it may be possible to model CRS in a xenograft model with intact recipient myeloid cells [186] and indeed, xenograft models have played an important role in helping to establish the role of GM-CSF in CAR T-cell toxicity [71], for example, despite their limitations.

Immunocompetent mouse models have been valuable in improving upon some of the deficiencies inherent in xenograft models and several unique systems have been employed in CAR T-cell toxicity research. Syngeneic models permit the study of toxicity in the presence of an intact (albeit often antigen inexperienced) host immune system using murine-derived tumor cell lines and CAR T-cells [187, 188]. Alternatively, transgenic mouse models can also permit the study of toxicity in an immunocompetent setting, such as the model used by Pennell et al. in which mice engineered to express human-CD19 and engrafted with a transgenic human-CD19-expressing lymphoma were treated with human-CD19 specific murine CAR T-cells to study toxicity mechanisms [189]. However, these models are often costly and may suffer from breeding challenges, making these models hard to sustain. Additionally, humanized mouse models that allow for the engraftment of human peripheral blood mononuclear cells (PBMCs) or hematopoietic stem cells (HSCs) into immunodeficient mice may help to provide a more representative model of the interaction between CAR T-cells and a human immune system. Despite potential difficulties with immune reconstitution, humanized models have been helpful in establishing the role of key cytokines such as IL-1 and IL-6 in toxicity as well as modeling unique toxicity mitigation strategies [190].

Aside from murine models, some have endeavored to take advantage of the homology between humans and nonhuman primates to study CAR T-cell toxicity. Taraseviciute et al. successfully modeled CRS and ICANS in a nonhuman primate (NHP) model of B-cell-directed CAR T-cell therapy for the first time [191]. Notably, this work demonstrated both CAR and non-CAR T-cell infiltration into the CNS along with increased proinflammatory cytokines in the CNS compared to serum, findings that could help to shed light on the obscure pathophysiology of ICANS in humans. Others have demonstrated the utility of this NHP model in studying on-target/off-tumor toxicity [192, 193]. The potential to study toxicities in the presence of an intact immune system in a species that more closely resembles humans makes this an important approach for those able to navigate the logistics and resources required for its implementation.

In all, while no perfect system for modeling CAR T-cell toxicity in humans exists, various systems with unique advantages and disadvantages have fostered improvement in our understanding and have provided a foundation for the development of novel methods of toxicity mitigation.

### Preclinical advances in toxicity mitigation

Various promising preclinical attempts to engineer methods of mitigating CAR T-cell toxicity while preserving efficacy have been put forth using the modeling systems discussed above. Whether it be modification of the CAR construct itself, unique gating approaches, or implementation of suicide switches, several approaches deserve discussion [194].

One simple approach has been to adjust the affinity of the CAR binding domain for its target antigen to tune CAR responsiveness to antigen density. Malignant cells often display higher antigen densities than nonmalignant cells, thus a lower binding affinity could facilitate discrimination in favor of recognition of higher-density malignant cells [195]. Indeed, higher affinity CAR constructs without this ability to discriminate have the potential to lead to devastating on-target/off-tumor effects [84]. The generation of lower affinity constructs may result in the selective targeting of malignant tissue in the context of certain target antigens [196–199] and encouragingly, the feasibility and reasonable toxicity profile of low-affinity CAR T-cells have been demonstrated in several clinical trials [200–202]. An additional challenge, especially for CAR T-cells for solid tumors, is suboptimal CAR T-cell trafficking to the tumor site, which could result in more CAR T-cells encountering healthy tissues expressing the target antigen. Engineering CAR T-cells to coexpress chemokine receptors, such as CXCR1 or CXCR2 for IL-8-secreting ovarian or pancreatic tumors, may be an additional strategy to direct cells to the tumor and mitigate on-target/off-tumor toxicity in other tissues [203].

Logic-gating techniques incorporating two or more receptors are designed to promote the preferential targeting of tumor cells [204]. For example, some designs require the recognition of two target antigens by separate CARs for optimal T-cell signaling and activation through the dissociation of signaling domains [205–210]. Alternatively, the synNotch system in which a synthetic Notch receptor recognizes its target antigen and induces the expression of a CAR that, upon recognition of a second target antigen, activates T-cell signaling has been described [211–216]. Both systems are designed to restrict CAR T-cell activation to tissues in which two targets are expressed, sparing healthy tissue that may only express one or the other. Conversely, the incorporation of an inhibitory receptor along with a CAR has been studied to prevent CAR T-cell activation upon recognition of an antigen expressed on healthy tissue [217, 218]. For example, Fedorov et al. described a system using a PD1- or CTLA4-based inhibitory receptor in conjunction with an activating CAR to hone CAR T tissue specificity. Despite the development of numerous inhibitory strategies and some early preclinical efficacy, there has been a lack of translation to the clinic thus rendering them an unproven method for toxicity mitigation at present.

Suicide switches have gained much interest in part related to their potential to provide an adjustable check on CAR T-mediated toxicities. Numerous strategies have been described and are detailed elsewhere [219–221]. One broad approach has been using antibody-mediated CAR destruction, such as through targeting of CD20 epitopes incorporated into the CAR with rituximab [222–224], or using cetuximab to target CARs expressing truncated EGFR [225–227]. One potential pitfall with these approaches is the necessity to achieve sufficient antibody concentrations for complete cell clearance, which may not be achieved across all tissues or be effective in patients who are leukopenic and the mechanism of action is relying on antibody-dependent cellular cytotoxicity [228–230]. Additionally, inducible caspase 9 (iCasp9) [231–234] or Fas [235] domains can be incorporated and activated to induce apoptosis upon the introduction of a small molecule and have shown promise in certain adoptive cell therapy platforms. Other methods of small molecule regulation of CAR expression, such as ligand-induced degradation [236], have the potential to allow for adjustable and reversible tuning of CAR T-cell activity in the presence of toxicity [165–168, 237].

Numerous other strategies, including modulating cytokine production [169, 238], honing responses to a tumor-specific milieu [239, 240], novel cell platforms [170, 171], or modulation of target antigen expression on healthy tissue [172] (NCT04849910), are also strategies that have been or are being explored for CAR T-cell-associated toxicity mitigation.

### Clinical advances in toxicity mitigation

Beyond the preclinical advances described above, several important strides have been taken in clinical management as well. Given the important role of IL-1 in CAR T-cell cytokine-mediated toxicities and preclinical evidence that IL-1 blockade can ameliorate these effects [186, 190], the anti-IL-1 monoclonal antibody anakinra has been studied clinically and shown utility in the management of refractory CRS and ICANS [177, 241]. Ongoing efforts have also begun to investigate the potential use of anakinra as a prophylactic agent [179]. Similarly, the success of IL-6 blockade with tocilizumab, which inhibits the IL-6 receptor, has led to interest in the use of the direct IL-6 inhibitor siltuximab [180, 181, 242], particularly given the ineffectiveness of tocilizumab in management of ICANS, the concern for a paradoxical increase in circulating IL-6 after tocilizumab administration [243], and the subsequent elevation of IL-18 following administration in the setting of IEC-HS [244]. Emapalumab, a monoclonal antibody targeting IFNγ, has shown preclinical efficacy in toxicity mitigation as well [245, 246]. Anecdotal evidence suggests a potential clinical utility [247]; however, further clinical validation is needed. As previously discussed, various other agents such as ruxolitinib and etoposide have also been used in specific clinical settings, such as IEC-HS, and continue to be investigated as mitigating agents. As mentioned above, the role of GM-CSF in cytokine-mediated toxicities has resulted in interest in its blockade [71, 248, 249] and an ongoing evaluation of lenzilumab, a GM-CSF inhibitor, is the subject of an ongoing early phase clinical trial (NCT04314843). The ability of the tyrosine kinase inhibitor dasatinib to interfere with T-cell signaling has led to interest in its use as a reversible suppressor of CAR T-cell proliferation and cytokine production [250, 251]. However, to date clinical results are limited [252] and further study is needed.

## Conclusion

With now over a decade of clinical experience, many CAR-associated toxicities such as CRS and ICANS are well-characterized entities with generally accepted management strategies. However, as CAR T-cell therapy expands to a wider pool of patients utilizing unique targets and innovative engineering techniques, new toxicities (such as IEC-HS and TIAN) have and will continue to emerge, emphasizing the need for ongoing research efforts directed at characterizing and mitigating toxicity. Additionally, even with the improvement in supportive care measures, CAR-associated toxicity is still a significant cause of morbidity and mortality, often necessitating ICU-level care and highlighting the importance of collaboration with critical care colleagues as well as supportive interventions specifically tailored to this unique ICU population. With improvement in preclinical modeling and a mechanistic understanding of toxicity, strategies for toxicity mitigation are rapidly evolving and the paradigm by which we manage CAR-associated toxicity will undoubtedly evolve as well. Future efforts will need to focus on thoughtful and tailored translation of novel approaches to the clinic as well the incorporation of alternate clinically available agents to improve upon existing management standards. Ultimately, optimized approaches to prevention and treatment of CAR-associated toxicity will continue to allow clinicians to safely deliver this revolutionary therapy to all who may benefit.

## Data Availability

No datasets were generated or analysed during the current study.
